# Multi-Omics Data Integration Reveals Sex-Dependent Hippocampal Programming by Maternal High-Fat Diet during Lactation in Adult Mouse Offspring

**DOI:** 10.3390/nu15214691

**Published:** 2023-11-05

**Authors:** Thibaut Gauvrit, Hamza Benderradji, Alexandre Pelletier, Soulaimane Aboulouard, Emilie Faivre, Kévin Carvalho, Aude Deleau, Emmanuelle Vallez, Agathe Launay, Anna Bogdanova, Mélanie Besegher, Stéphanie Le Gras, Anne Tailleux, Michel Salzet, Luc Buée, Fabien Delahaye, David Blum, Didier Vieau

**Affiliations:** 1 UMR-S1172, Lille Neurosciences & Cognition, University of Lille, INSERM, CHU Lille, 59000 Lille, France; thibaut.gauvrit@inserm.fr (T.G.); hamza.benderradji@chu-lille.fr (H.B.); emilie.faivre@inserm.fr (E.F.); kevin.carvalho.fr@gmail.com (K.C.); 1rohmera@gmail.com (A.D.); agathe.launay@inserm.fr (A.L.); anna.bogdanova@inserm.fr (A.B.); luc.buee@inserm.fr (L.B.); david.blum@inserm.fr (D.B.); 2 Alzheimer & Tauopathies, LabEX DISTALZ, 59045 Lille, France; 3 The Department of Pharmacology & Biophysics, Chobanian & Avedisian School of Medicine, Boston University, Boston, MA 02118, USA; admpelletier@gmail.com; 4 U1192—Laboratoire Protéomique, Réponse Inflammatoire et Spectrométrie de Masse (PRISM), University of Lille, INSERM, 59000 Lille, France; soulaimane.aboulouard@univ-lille.fr (S.A.); michel.salzet@univ-lille.fr (M.S.); 5 Institut Pasteur de Lille, U1011-EGID, University of Lille, INSERM, CHU Lille, 59000 Lille, France; emmanuelle.vallez@pasteur-lille.fr (E.V.); anne.tailleux@univ-lille.fr (A.T.); 6 US 41-UMS 2014-PLBS, Animal Facility, University of Lille, CNRS, INSERM, CHU Lille, 59000 Lille, France; melanie.besegher@univ-lille.fr; 7 CNRS U7104, INSERM U1258, GenomEast Platform, IGBMC, University of Strasbourg, 67412 Illkirch, France; stephanie.le-gras@igbmc.fr; 8 Sanofi Precision Medicine and Computational Biology, 94081 Vitry-sur-Seine, France; fabien.delahaye@sanofi.com

**Keywords:** maternal high-fat diet, multi-omics, sexual dimorphism, hippocampus

## Abstract

Early-life exposure to high-fat diets (HF) can program metabolic and cognitive alterations in adult offspring. Although the hippocampus plays a crucial role in memory and metabolic homeostasis, few studies have reported the impact of maternal HF on this structure. We assessed the effects of maternal HF during lactation on physiological, metabolic, and cognitive parameters in young adult offspring mice. To identify early-programming mechanisms in the hippocampus, we developed a multi-omics strategy in male and female offspring. Maternal HF induced a transient increased body weight at weaning, and a mild glucose intolerance only in 3-month-old male mice with no change in plasma metabolic parameters in adult male and female offspring. Behavioral alterations revealed by a Barnes maze test were observed both in 6-month-old male and female mice. The multi-omics strategy unveiled sex-specific transcriptomic and proteomic modifications in the hippocampus of adult offspring. These studies that were confirmed by regulon analysis show that, although genes whose expression was modified by maternal HF were different between sexes, the main pathways affected were similar with mitochondria and synapses as main hippocampal targets of maternal HF. The effects of maternal HF reported here may help to better characterize sex-dependent molecular pathways involved in cognitive disorders and neurodegenerative diseases.

## 1. Introduction

The prevalence of obesity has been rising steadily for several decades, and is predicted to reach 18% in men and surpass 21% in women in 2025, three times more than in 1975 [1,2]. As a result, the proportion of overweight or obese childbearing women has also risen, suggesting that children are exposed to high-calorie food during the early phases of their development [3]. Although wealthy countries are the most affected, with obesity affecting almost half of pregnant women in the United States [4], low- and middle-income countries are also impacted, making obesity in pregnancy a global health issue [5]. In addition to being a risk factor for many disorders for pregnant women [6], maternal obesity can also have consequences for offspring. Indeed, it is now widely accepted that maternal obesity can have long-term consequences for the adult offspring, as stipulated by the concept of the developmental origins of health and disease (DOHaD), introduced in the 1980s by David Barker [7]. This concept, also known as perinatal programming, suggests that environmental stress during the perinatal period, such as maternal food overconsumption, modifies the developmental trajectory of the fetus and/or the newborn and diminishes plasticity reserves, which may have a long-lasting effect increasing the risk of developing diseases in adulthood [8,9,10,11].

Since this pioneering work [12], numerous epidemiological studies in humans have confirmed that maternal obesity might promote the development of cardiometabolic disorders in adult offspring [13]. To better understand the underlying biological mechanisms, researchers have developed animal models that mimic maternal obesity, notably by feeding females with high-fat diets (HF) before and/or during gestation and/or lactation [14]. Although most of these studies have focused on metabolism, recent epidemiological studies have reported that maternal obesity can also lead to neurodevelopmental alterations in the offspring, which may increase the risk of developing cognitive disorders. Indeed, a recent meta-analysis indicates that maternal obesity before pregnancy increases the risk of attention deficit-hyperactivity disorder, autism spectrum disorder, developmental delay, and emotional/behavioral problems in child [15].

Although these reports focused on the consequences for adolescents due to the difficulty of longitudinal follow-up, several experimental studies using different animal models suggest that maternal HF during the perinatal period may increase the risk of locomotor, memory, and anxiety disorders at later stages in older animals [16,17]. These few experimental studies, mainly performed in the hippocampus, a brain structure particularly sensitive to metabolic stress [18] and playing a crucial role in behavioral processes, have associated cognitive disorders with synaptic modifications [19], neuroinflammation [20,21], and metabolic impairments [22]. However, the data remain sometimes contradictory and further work is necessary to determine the underpinning biological mechanisms. Indeed, while most studies show that maternal HF induces impaired cognition associated with synaptic loss [22,23], it has also been reported that a similar diet improves memory and synaptic plasticity [24]. Most of these studies have been performed when cognitive disorders and brain alterations are clearly identified. It therefore seems interesting to look at the consequences at earlier stages. Moreover, although sex-dependent consequences of obesogenic maternal diets have been reported in the offspring, most studies have been usually performed on a single sex [21,25,26,27,28]. Thus, in the current study, we explored the early consequences of maternal HF both in male and female young adult mouse offspring before the appearance of significant metabolic and cognitive dysfunctions. Maternal HF was applied during lactation, a critical-time window corresponding to the peak of brain growth in rodents, which is very sensitive to environmental perturbations [29,30]. This critical developmental time period corresponds with the third trimester of gestation and the very beginning of lactation in humans, when synaptogenesis, axon maturation, dendritic growth, and gliogenesis are taking place [29,31]. To identify the early cellular and molecular hippocampal targets of maternal HF in the offspring, we have carried out an unsupervised approach using a multi-omics strategy (transcriptomics, proteomics, and regulomics). This type of strategy has already been successfully used to study modifications during aging that are associated with cognitive impairments, and has enabled the discovery of new biological pathways [32,33]. However, to our knowledge no such strategy has been used to decipher the early hippocampal consequences of maternal HF in male and female mouse offspring.

## 2. Materials and Methods

### 2.1. Animals

**Figure 1:**
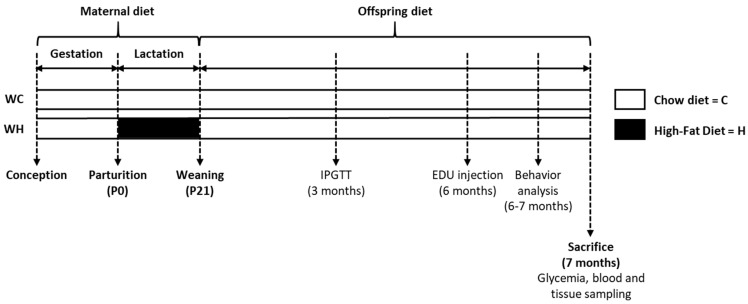
Experimental protocol. C57Bl6/J (W) dams received either a chow (13.5% of fat, C; *n* = 14 dams) or high-fat (58% of fat, H; *n* = 14 dams) diet during lactation generating 2 groups: WC and WH offspring. After weaning, offspring fed a chow diet (8.4% of fat) until sacrifice at 7 months of age (*n* = 19 WC females; *n* = 19 WH females; *n* = 17 WC males; *n* = 17 WH males). An intraperitoneal glucose tolerance test (IPGTT) was performed at 3 months of age. 5-ethynyl-2′-deoxyuridine (EDU) injection and behavioral analysis were done respectively, at 6 and 7 months of age.

Eight-week-old C57Bl6/J female mice (W) were mated with THY-Tau22 (T) male mice, a heterozygous model of tauopathy (2 females per male). After 2 weeks, females were housed individually. During lactation (postnatal day P0 to P21), females were randomly allocated to either a high-fat (58% of fat, Research Diets D12331; H; *n* = 14 dams) or a control diet (13.5% of fat, Safe A03; C; *n* = 14 dams), generating 2 groups: WC and WH mice. Nutrient composition of each diet is listed in Supplementary Table S1. At P1, litter size was adjusted to 6 pups per dam. At weaning (P21), offspring was fed a standard diet (8.4% of fat, Safe A04) and housed according to sex and maternal diet (3 animals per cage) until the sacrifice at 7 months of age (*n* = 19 WC females; *n* = 19 WH females; *n* = 17 WC males; *n* = 17 WH males). In all groups body weight, food and water intake were recorded weekly (Figure 1).

All animals were housed in a pathogen-free facility (University of Lille) and maintained under conditions controlled for temperature (22 °C and 12-h light/12-h dark cycle) with ad libitum access to food and water. All protocols were approved by an ethics committee (protocol #8677-2017011213553351v3).

### 2.2. Behavioural Study

Behavioral experiments were performed in animals between 6 and 7 months of age, randomly assigned by experimenters blinded to the genotype and phenotype of mice. Before testing the memory of mice, we made sure that they had no locomotor or anxiety impairment that might introduce bias in the analysis of the memory tests (actimetry, elevated plus maze; Supplementary Material). Long-term spatial memory was evaluated using the Barnes maze task. This maze is an elevated white circular PVC open platform (80 cm height, 120 cm diameter) with 40 equally spaced holes (5 cm of diameter) located at 5 cm from its circumference and a black escape box located under one of the 40 holes. This platform is surrounded by spatial cues to help guide the animal to the escape hole. To encourage the mouse to enter the escape box, a bright light was placed over the maze (900 lux). First, for the habituation step, mice were free to explore the maze and the escape box for 3 min. Next, mice completed 4 days of acquisition training with 4 trials per day. For each trial, mice were placed in the start tube in the center of the maze (for 5 s) and then trained to locate the escape hole (randomized for all mice) using spatial cues surrounding the maze. If a mouse did not enter the escape hole in 3 min it was gently guided to the escape hole. Mice remained 60 s in the escape box before returning to their home cage. The inter-trial interval was 15 min. After each test, the maze was cleaned with 70% ethanol to avoid odor bias and the maze was rotated clockwise a quarter turn every day. For each trial, distance travelled, velocity, and time to find the target hole (latency) are measured by Ethiovision XT tracking system (Noldus). The retention step takes place 72 h after the last test. Each mouse was placed on the maze without the escape box for 2 min. Time spent in each quadrant was measured. Mice exploring the maze for less than 30 s were excluded (1 WC and 1 WH male mice).

### 2.3. Sacrifice and Brain Tissue Preparation

Animals were sacrificed at 7 months of age, after 6 h of fasting. Mice were euthanized by cervical dislocation. Brains were removed, hippocampi were dissected before being frozen in dry-ice and stored at −80 °C for biochemical and RNA analyses. A subgroup of 7-month-old mice injected with 5-ethynyl-2′-deoxyuridine (EDU; 900584, Sigma-Aldrich, Saint-Louis, MO, USA) (see Section 2.7 for the protocol) were deeply anaesthetized with pentobarbital sodium and perfused with cold saline solution 0.9% and paraformaldehyde (PFA) 4% (*n* = 7 WC females; *n* = 7 WH females; *n* = 4 WC males; *n* = 8 WH males). Brains were removed and post-fixed in PFA 4% for 24 h and incubated in a sucrose 30% solution for 2 days, before being frozen in 2-methyl-butane at −40 °C for 1 min and stored at −80 °C.

### 2.4. Glycaemia

Blood glucose measurements in mice were performed after 6 h of fasting (postabsorptive condition) at 7 months of age (at the time of sacrifice). A drop of blood, obtained after incision of the tail vein was applied to a OneTouch strip linked to a OneTouch Verio Flew glucometer.

### 2.5. Glucose Tolerance Test

Glucose tolerance was assessed at 3 months of age using the intraperitoneal glucose tolerance test (IPGTT) following 6 h of morning fasting. D (+) glucose (1 g/kg; G8270, Sigma-Aldrich) was injected intraperitoneally. Blood glucose was then measured (see Section 2.4. for the protocol) at 0, 15, 30, 60, 90 and 120 min following injection.

### 2.6. Biochemical Plasma Parameters

Blood was collected at the tail vein after 6 h of fasting just before the animals were sacrificed (7 months of age). Blood was centrifuged at 1500× *g* for 15 min at 4 °C. Plasma was separated, transferred to 1.5 mL Eppendorf tubes, and stored at −80 °C until analysis. Plasma concentration of insulin was measured using the mouse insulin ELISA kit (10-1247-01, Mercodia AB; no cross-reactivity with proinsulin) following the manufacturer’s instructions. Commercially available kits were used to measure total cholesterol (TC) (MG981813, Thermo Fischer Scientific, Waltham, MA, USA), triglycerides (TG) (157109910026, Diasys, Holzheim, Germany) and free fatty acids (FFA) (157819910935, Diasys) plasma concentrations applied to a biochemistry analyzer (Konelab20, Thermo Fischer Scientific) with colorimetric methods.

### 2.7. Adult Hippocampal Neurogenesis Analysis

#### 2.7.1. 5-Ethynyl-2′-Deoxyuridine (EDU) Injection

In order to study the neurogenesis, 6-month-old mice (*n* = 7 WC females; *n* = 7 WH females; *n* = 4 WC males; *n* = 8 WH males) were injected with EDU (900584, Sigma-Aldrich), a modified thymidine analog that incorporates into DNA during cell division. Fifty mg/kg of body weight of EDU were injected 2 times per day for 3 days, 21 days before the sacrifice.

#### 2.7.2. EDU Staining

The brains were cut to obtain 35 µm coronal floating sections using a cryostat. EDU was fluorescently labeled thanks to the Click-iT EDU 647 nm kit following the manufacturer’s instructions (BCK-EDU647, Sigma-Aldrich). EDU staining was performed on serial sections of 35 µm separated by 350 µm. The images were obtained using an axioscan microscope (Axioscan Z1, Zeiss, Oberkochen, Germany) with a 20X objective. The number of EDU-positive cells in the subgranular zone of the hippocampal dentate gyrus was counted manually on serial coronal sections along the hippocampus.

### 2.8. RNA-Sequencing Analysis

#### 2.8.1. RNA Extraction

Total RNA was extracted from hippocampal tissues using RNeasy Lipid Tissue Mini kit (1023539, QIAGEN; *n* = 4/group for males and *n* = 5/group for females). Freshly dissected tissue was disrupted using a polytron (PT2500E, Kinematica, Malters, Switzerland), homogenized in 900 µL of QIAzol lysis reagent, before chloroform extraction. After centrifugation (12,000× g, 15 min at 4 °C), the supernatant is recovered and RNA was precipitated with 70% ethanol, and transferred in RNeasy Mini spin column, washed and eluted in 40 µL RNase-free water. Quantity and quality were checked using a fluorometer (Qubit, ThermoFisher Scientific) and a capillary electrophoresis machine (Bioanalyzer, Agilent, Santa Clara, CA, USA). All samples have an RNA integrity number above 7, a ratio 28S/18S above 1.6, and sufficient RNA quantity for RNA-sequencing.

#### 2.8.2. RNA-Sequencing Analysis

RNA-sequencing (RNA-seq) libraries were generated (*n* = 5 for females and *n* = 4 for males from each group) from 300 ng of total RNA using TruSeq Stranded mRNA Library Prep Kit (20020595, Illumina, San Diego, CA, USA) and IDT for Illumina—TruSeq RNA UD Indexes (96 Indexes, 96 Samples) (20040871, Illumina), according to manufacturer’s instructions. Briefly, following purification with poly-T oligo attached magnetic beads, the mRNA was fragmented using divalent cations at 94 °C for 8 min. The cleaved RNA fragments were copied into first-strand cDNA using reverse transcriptase and random primers. Strand specificity was achieved by replacing dTTP with dUTP during second strand cDNA synthesis using DNA Polymerase I and RNase H. Following the addition of a single ‘A’ base and subsequent ligation of the adapter on double-stranded cDNA fragments, the products were purified and enriched with PCR [30 s at 98 °C; (10 s at 98 °C, 30 s at 60 °C, 30 s at 72 °C) × 12 cycles; 5 min at 72 °C] to create the cDNA library. Surplus PCR primers were removed by purification using SPRI select beads (wsr-410564, Beckman-Coulter, Brea, CA, USA) and the final cDNA libraries were checked for quality and quantified using capillary electrophoresis [average size ranging from 200 to 600 bp and limited presence of adapter dimers (120–130 bp band)]. Sequencing was performed on the Illumina Genome HiSeq 4000 and NextSeq 2000 as single-end 50-base reads following manufacturer’s instructions.

#### 2.8.3. Data Processing

Cutadapt 1.10 (--anywhere “A{100}” --quality-cutoff 20,20 --adapter AGATCGGAAGAGCACACGTCTGAACTCCAGTCAC --minimum-length 40) was used to trim adapter and low-quality (Phred quality score bellow 20) bases and remove reads shorter than 40 bp after trimming. Reads mapping to rRNA sequences were also discarded. Reads were then mapped onto the mm10 assembly of Mus musculus genome using STAR version 2.5.3a (--twopassMode Basic) [34]. Gene expression was quantified using htseq-count release 0.6.1p1 (--mode union --minaqual 10) [35] and gene annotations from Ensembl release 102. Statistical analysis was performed using R and DESeq2 1.16.1 Bioconductor library [36].

From expression matrix, gene set enrichment analysis was performed using GSEA software version 4.3.2 available on www.gsea-msigdb.org. We ran our expression dataset against a library of 10,673 ontology gene sets (7842 biological process, 1028 cellular component, 1803 molecular function; m5.all.v2022.1.Mm.symbols.gmt). The statistical significance (nominal *p* value) of the enrichment score (ES) was estimated by running 1000 gene set permutations. The ES was normalized (NES) to account for the size of the gene set. To adjust for multiple testing across the 10,673 gene sets, we computed the false discovery rate (FDR) and controlled the FDR at 25%, which corresponds to the threshold recommended in the GSEA user guide. Gene sets enrichment network was visualized using cytoscape software version 3.9.1. Clusters of gene sets were automatically annotated using spectral clustering of protein sequences (SCPS) cluster algorithm (label column: name, label algorithm: adjacent words, 3 words per label). Clusters with less than 5 set genes were removed to allow a better visualization. In addition, from GSEA results, we performed a leading edge analysis (LEA) of common mitochondria-related gene sets between males and females, to identify whether the genes most involved in enriching these gene sets are similar between both sexes. The genes that participate in the enrichment of at least 50% of the gene sets for LEA analysis for each sex were kept and considered as the most relevant genes.

Sequencing data that support the findings of this study have been deposited in the NCBI’s Gene Expression Omnibus (GEO) database (GSE167123).

### 2.9. Mass Spectrometry

#### 2.9.1. Protein Extraction

Hippocampal proteins were extracted from frozen tissue (−80 °C). Hippocampal tissue was sonicated on ice in 200 µL of Tris buffer containing 10% sucrose (pH 7.4) and homogenized at 4 °C for 1 h using a rotating mixer (714-459-0784, Benchmark). Homogenates were kept at −80 °C until use. Protein concentrations were quantified using a bicinchoninic acid (BCA) protein assay (1023539, Thermo Fisher Scientific, Waltham, MA, USA). Briefly, 25 µL of 1:10 diluted sample and a range of bovine serum albumin (BSA) from 0 to 2000 µg/mL were loaded to a 96-well plate. Then, 200 µL of reagent (196 µL reagent A + 4 µL reagent B) were added. After 30 min at 37 °C, absorbance at 570 nm was measured using a photometer (multiskan FC, Thermo Fisher Scientific).

#### 2.9.2. Proteomic Preparation

A shotgun bottom-up proteomic approach was carried out. All samples were resuspended in 100 µL of ammonium bicarbonate buffer (NH_4_HCO_3_, 50 mM) and 50 µL of denaturation buffer (urea 6 M) were added, and the mixture was incubated and sonicated for 5 min. After denaturation, 50 µL of reduction buffer (Dithiothreitol- DTT 40 mM) were added to the sample, and incubated at 56 °C for 40 min. Next, 50 µL of alkylation buffer (Iodoacetamide IAA 100 mM) were added to the sample, and the mixture was incubated in darkness for 40 min to allow for alkylation. To neutralize the free IAA, 50 µL of 600 mM Thio-urea buffer were added to the samples. A subsequent addition of 200 µL of ammonium bicarbonate buffer was made to reduce the urea concentration to below 1 M. For enzymatic digestion, a final concentration of 3% trypsin (3 µg trypsin/100 µg protein) was added to the diluted samples, and the mixture was incubated overnight at 37 °C. To stop the digestion process, 10 µL of 5% trifluoroacetic acid (TFA) were added to the digested sample. Finally, the samples were dried using a SpeedVac. Prior to nLC-MS/MS analysis, the samples were desalted using ZipTip C-18 (ZTC18S096, Millipore, Burlington, MA, USA).

#### 2.9.3. NanoLC-MS/MS Analysis

The digested proteins were analyzed utilizing a Nano Acquity UPLC system (Waters) connected to a Q-Exactive Orbitrap mass spectrometer (ThermoFisher Scientific) through a Nanospray Flex source. The samples were separated by means of an online reversed phase, using a pre-concentration column (nanoAcquity Symmetry C18, 5 µm, 180 µm × 20 mm) and an analytical column (nanoAcquity BEH C18, 1.7 µm, 75 µm × 250 mm). The peptides were separated by applying a linear gradient of acetonitrile in 0.1% formic acid (5–30%) for 2 h, at a flow rate of 300 nl/min. The Q-Exactive was operated in data-dependent acquisition (DDA) mode defined to analyze the ten most intense ions of MS analysis (Top 10). The MS analysis was performed with a m/z mass range between 300 and 1600, resolution of 70,000 FWHM, AGC of 3e6 ions and maximum injection time of 120 ms. The MS/MS analysis was performed with a m/z mass range between 200 to 2000, AGC of 5e4 ions, maximum injection time of 60 ms and resolution set at 17,500 FWHM. Higher energy collision dissociation (HCD) was set to 30%. Precursors ions with charges states >+1 and <+8 were kept for the fragmentation, with a dynamic exclusion time of 20 s.

#### 2.9.4. Data Processing

The proteins were identified by comparing all MS/MS data with the proteome database of Mus musculus (Uniprot, release December 2021, 86,492 entries), using the MaxQuant software version 2.0.3.0 [37]. The oxidation of methionine and N-terminal protein acetylation were defined as variable modifications. The carbamidomethylation of cysteine was chosen as a fixed modification. Label-free quantification (LFQ) was done keeping the default parameters of the MaxLFQ algorithm [38]. The digestion parameters were defined using trypsin with 2 maximum missed cleavages. The protein and peptide identification parameters were carried out with a false discovery rate (FDR) of 1%, and a minimum of 2 peptides per protein including 1 unique. Next, the analysis was conducted by Perseus software (https://maxquant.net/perseus/, accessed on 15 April 2023) version 1.6.10.43 [39]. The LFQ intensity of each sample was downloaded in Perseus and the data matrix was filtered by removing the potential contaminants, reverse and only identified by site. The data were then transformed using the log2(x).

The mass spectrometry proteomics data have been deposited to the ProteomeXchange Consortium via the PRIDE [40] partner repository with the dataset identifier PXD045639.

### 2.10. Regulon Analysis

To analyze the transcription factor (TF) activity in maternal high-fat vs. chow diet mice, we leveraged both transcriptomics and proteomics data using co-expression network analysis and TF motif analysis. Similarly to the SCENIC R package designed for TF module analysis in single cell data [41], we used the package R GENIE3 using random forest machine learning method to infer regulatory link [42] and the CisTarget database to perform TF motif enrichment [41] and keep TF-gene links with evidence of direct interaction.

Briefly, the transcriptomics data were filtered to conserved only genes with more than 1 count per million reads in at least 10% of the samples, and the proteomics data were filtered to conserved only proteins without missing values and then transform using log2. Mice TFs were annotated thanks to the TRRUST database v2 [43]. Twenty mice samples were shared between transcriptomics and proteomics data and then used for downstream analysis. GENIE3 function was used to calculate the regulatory link score between TF and gene, based on TF abundance and gene expression correlation. Each regulatory link was then assigned a sense (positive or negative regulation) using the sign of the spearman correlation coefficient. Only TF-gene links with a link score of more than 0.02 were conserved and five candidates modules based on different filtering threshold were created by TF: (i) keeping all TF-gene pairs with a regulatory score greater than 0.02; (ii) keeping all TF-gene pairs with a regulatory score greater than 0.05; (iii) keeping the top 50 genes by TF; and (iv) keeping the top 5 TF by gene targets or (v) keeping the top 10 TF by gene targets.

TF motif enrichment was then performed on these TF modules candidates using the fgsea package [44] and the mouse musculus motif ranking (v10) of the CisTarget database. We used the motifs ranking spanning a 10 kb window around TSS (available here https://resources.aertslab.org/cistarget/databases/mus_musculus/mm10/refseq_r80/mc_v10_clust/gene_based/mm10_10kbp_up_10kbp_down_full_tx_v10_clust.genes_vs_motifs.rankings.feather, accessed on 13 June 2023). Only TF modules candidates with a corresponding TF motif enrichment at adjusted *p*-value < 0.05 were conserved and were then filtered to keep only the leading edges genes for each TF motif. All enriched and filtered modules were merged by TF to obtain the final TF regulon. To confirm relevance of this approach to infer direct TF-gene interaction, we used a publicly available mouse hippocampus ChIP-seq data for Mecp2 regulon (GSE71126_RAW). We found that the ChIP-seq signal value of Mecp2 (binding intensity) was significantly greater (adjusted *p*-value < 0.001, rank-sum Wilcoxon test) in genes of the Mecp2 regulon than in other genes (data available at https://github.com/AlexandrePelletier/HippoProgRegulome, accessed on 10 September 2023).

Finally, the fgsea package was used to assess the enrichment of TF regulons in differentially expressed genes in maternal high-fat vs. chow diet mice. Only TFs regulons with |NES| > 2 and adjusted *p*-value < 0.05 were considered as positively or negatively enriched in maternal high-fat vs. chow diet mice. The enrichment of genes associated with these regulons was analyzed using the gene ontology (GO biological process, GO molecular function and GO cellular component) and the Kyoto encyclopedia of genes and genomes (KEGG) databases.

The R script is available at https://github.com/AlexandrePelletier/HippoProgRegulome, accessed on 10 September 2023.

### 2.11. Statistics

Image acquisition and quantification, as well as metabolic and behavioral evaluations, were performed by investigators blind to the experimental condition. Values are expressed as mean ± standard error of the mean (SEM). Normality of the distribution and homoscedasticity were checked using Shapiro–Wilk test and F or Bartlett tests, respectively. Differences between mean values were determined using two-tailed unpaired Student’s *t*-test, one sample *t*-test or two-way ANOVA followed by a post hoc Tukey’s multiple comparisons test using GraphPad Prism version 9.0.0 software. *p*-values < 0.05 were considered significant.

## 3. Results

### 3.1. Maternal High-Fat Diet Increases Body Weight Gain during Lactation in Offspring, and Induces a Glucose Intolerance in Adult Males

We evaluated the consequences of a maternal HF in both male and female offspring on physiological and metabolic parameters at 7 months of age before the onset of aging processes.

During lactation, the maternal HF decreases the body weight of dams associated with increased food intake (Supplementary Data S1) and increases the body weight of male and female offspring at P21 (males: *p* = 0.010, females: *p* = 0.001, unpaired *t*-test, Table 1). This increase is not sustained over time, as there is no significant change at 7 months of age and the body weight is even reduced in 7-month-old male offspring from HF mothers (*p* = 0.006, unpaired *t*-test). Similarly, there is no change in plasma metabolic parameters (free fatty acid, triglyceride, cholesterol), nor in glycaemia and body temperature. However, insulinemia after 6 h of fasting was significantly reduced by the maternal HF in 7-month-old male offspring (*p* = 0.030, unpaired *t*-test), but was not modified in females, suggesting sexual dimorphism in this parameter (Table 1).

### 3.2. Maternal High-Fat Diet Leads to Glucose Intolerance in a Sex-Dependent Manner in Offspring

**Figure 2:**
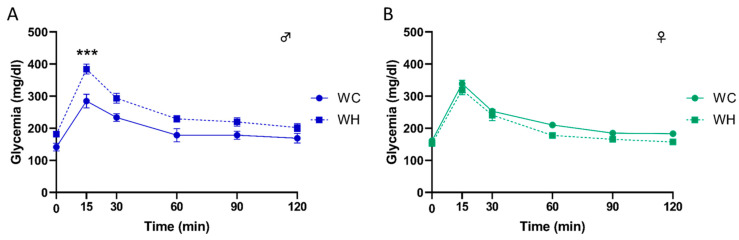
Effect of maternal high-fat diet during lactation on intraperitoneal glucose tolerance test (IPGTT) in 3-month-old male and female offspring. Glycaemia changes during IPGTT over time, respectively in males (**A**) and females (**B**). Values are represented as mean ± SEM. *** *p* < 0.001 vs. WC mice using two-way ANOVA followed by Tukey’s post hoc test. *n* = 19 WC females; *n* = 19 WH females; *n* = 17 WC males; *n* = 17 WH males.

To evaluate the impact of maternal HF on glucose metabolism, we challenged the 3-month-old mice with an intraperitoneal glucose tolerance test (IPGTT) and monitored glycaemia at different time points. Interestingly, the results indicate that the maternal HF induces a greater increase in glycaemia in male offspring at 15 min post-glucose injection, with a return to baseline at 120 min, suggesting a glucose intolerance (Figure 2A, *p* < 0.001 at 15 min following glucose injection, 2-way ANOVA followed by Tukey’s post hoc test). Furthermore, this effect was sex-dependent since it was not observed in females (Figure 2B).

### 3.3. Maternal High-Fat Diet Impairs Long-Term Spatial Memory in Offspring

**Figure 3:**
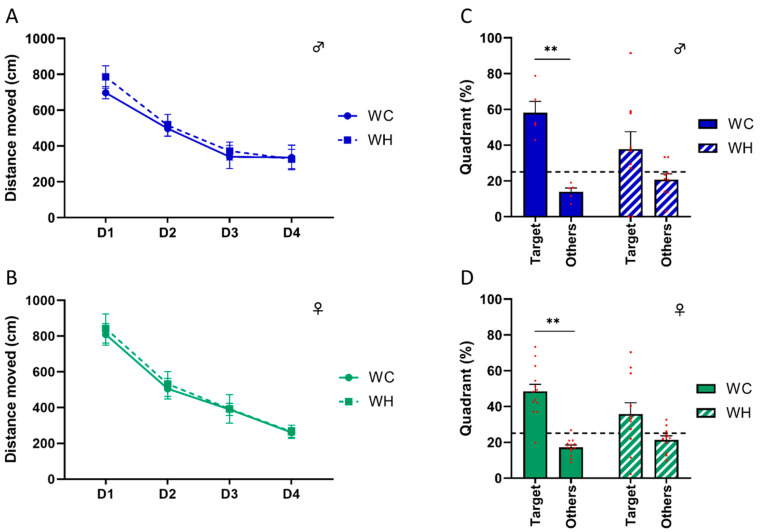
Effect of maternal high-fat diet during lactation on long-term spatial memory analysis using the Barnes maze task in 6-month-old male and female offspring. (**A**,**B**) Distance moved during the learning phase representing the capacity to find the escape box during the four days of training, respectively, in male and female offspring. (**C**,**D**) Percentages of time spent in the target and other quadrants during the probe test, 72 h after the last day of learning, respectively, in male and female offspring. The dotted line corresponds to the theoretical percentage chance of exploring the quadrant. Values are represented as mean ± SEM. ** *p* < 0.01 vs. WC using two-way ANOVA followed by Tukey’s post hoc test. *n* = 13 WC females; *n* = 11 WH females; *n* = 6 WC males; *n* = 10 WH males.

Although the aim of this study was to focus on stages before the onset of aging processes, including cognitive disorders, we tested the long-term spatial memory of 6-month-old mice. Indeed, some studies report cognitive impairment at early stages [22,45,46], although these results are still debated. Before studying the mice’s memory, we verified that the maternal HF did not alter the mice’s locomotion and anxiety, using the open field test and the elevated plus maze task, respectively. The data showed no difference, enabling us to carry out memory tests without bias (Supplementary Data S2). Next, we evaluated long-term spatial memory using the Barnes maze, which is a hippocampus-dependent task. During the learning phase, all groups showed a decrease in the distance moved across trials (*p* < 0.001, two-way ANOVA followed by Tukey’s post hoc test) (Figure 3A,B). Seventy-two hours following learning, a probe trial was performed to assess long-term spatial memory. Male and female WC mice explored the target quadrant significantly more than the other quadrants (males: *p* = 0.005, females: *p* < 0.001, two-way ANOVA followed by Tukey’s post hoc test), suggesting that they learned properly. Conversely, male and female mice from HF mothers did not explore the target quadrant significantly more than the other quadrants (males: *p* = 0.242, females: *p* = 0.064, two-way ANOVA followed by Tukey’s post hoc test) (Figure 3C,D). In line with this, only WH males and females explore the target quadrant more than 25% (chance level) of time (males: WC *p* = 0.006, WH *p* = 0.0224; females: WC *p* < 0.001, WH *p* = 0.1199; one sample *t*-test). These data indicate that the maternal HF impairs long-term spatial memory in adult offspring, suggesting hippocampal dysfunction in both sexes.

### 3.4. Maternal High-Fat Diet Increases the Number of Proliferating Neural Stem Cells in the Subgranular Zone in Male Offspring

**Figure 4:**
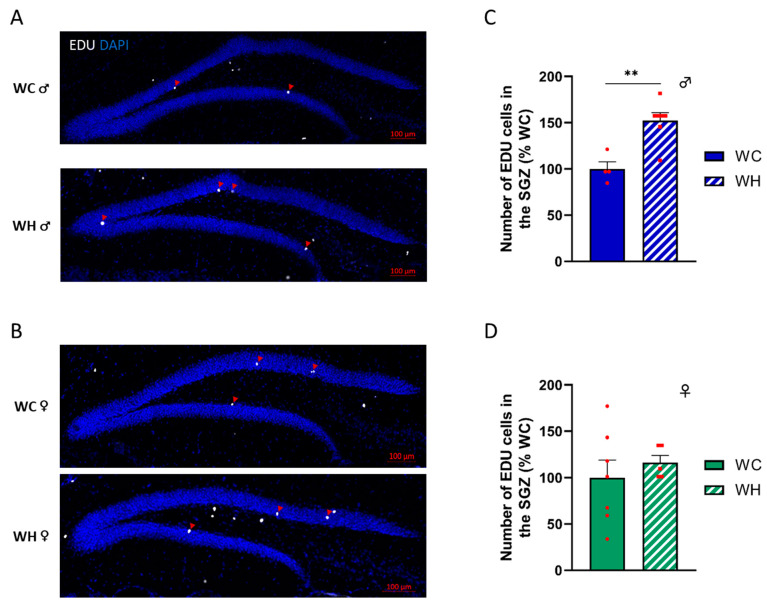
Effect of maternal high-fat diet during lactation on adult hippocampal neurogenesis of dentate gyrus cells in 7-month-old male and female offspring. (**A**,**B**) EDU incorporation in the cells of the subgranular zone (SGZ) of the dentate gyrus (red arrow), respectively, in male and female offspring. EDU was injected 21 days before sacrifice. (**C**,**D**) Quantification of the total number of proliferating cells in the SGZ (EDU positive) respectively, in male and female offspring. Values are represented as mean ± SEM. ** *p* < 0.01 vs. WC mice using Student’s unpaired *t*-test. *n* = 7 WC females; *n* = 7 WH females; *n* = 4 WC males; *n* = 8 WH males.

Past studies suggest that a maternal HF can modify adult hippocampal neurogenesis (AHN) [47], and that the dysregulation of AHN could lead to cognitive impairments [48]. To address this question, we injected intraperitoneally 5-ethynyl-2′-deoxyuridine (EDU), a modified thymidine analog that is incorporated into nascent DNA during cell division. The mice were sacrificed 21 days after the last injection, at 7 months of age. EDU^+^ cell count in the subgranular zone of the dentate gyrus of the hippocampus, the region where AHN takes place, indicates that the maternal HF induced an increase of AHN in male offspring (*p* = 0.002, unpaired *t*-test) (Figure 4A,C). In contrast, this effect was not observed in females, suggesting sexual dimorphism (Figure 4B,D).

### 3.5. Maternal High-Fat Diet Impairs Hippocampal Transcriptome in a Sex-Dependent Manner in the Offspring

**Figure 5:**
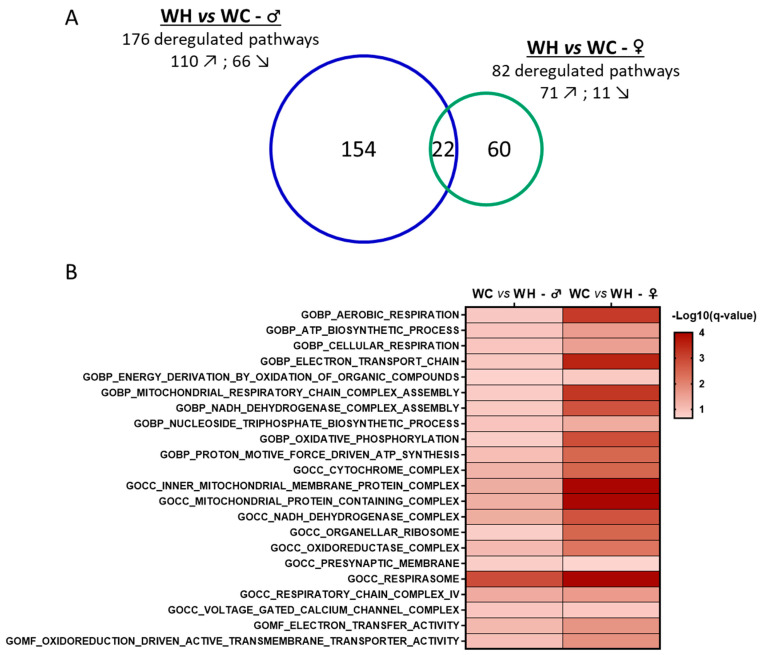
Sex comparison of gene set enrichment analysis (GSEA) results from the hippocampal transcriptome of 7-month-old male and female offspring. (**A**) The Venn diagram represents the number of gene ontology (GO) terms significantly enriched (false discovery rate < 0.25) in terms of cellular components, biological pathways and molecular functions in GSEA. (**B**) Heatmap of the 22 common significantly deregulated gene sets by the maternal high-fat diet between male and female offspring and associated significance. *n* = 5 WC females; *n* = 5 WH females; *n* = 4 WC males; *n* = 4 WH males. ↗ = pathways increased by the maternal high-fat diet. ↘ = pathways decreased by the maternal high-fat diet.

To identify early-deregulated biological pathways that may explain the observed cognitive alteration (Figure 3), we performed a hippocampal transcriptome analysis of 7-month-old offspring by RNA-seq. Gene set enrichment analysis (GSEA) reveals that the maternal HF leads to marked changes in the hippocampal transcriptome in both 7-month-old male and female offspring. In male offspring, among the 176 gene sets that were significantly modified by the maternal HF, 110 exhibited an activation whereas 66 showed an inhibition (Figure 5A). The effect of maternal HF in female offspring appears less prominent, with 82 gene sets significantly enriched, including 71 and 11 showing an activation and an inhibition by the maternal HF, respectively (Figure 5A).

**Figure 6:**
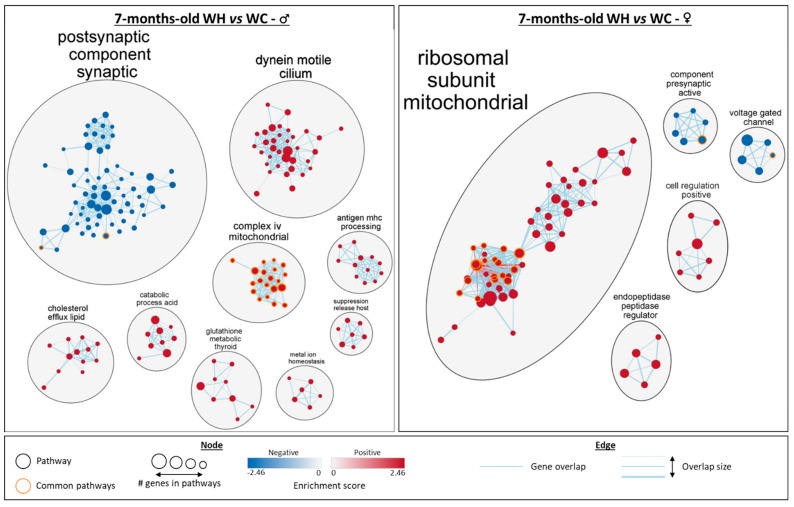
Network analysis of gene set enrichment analysis (GSEA) results from the hippocampal transcriptome of 7-month-old male and female offspring. The network, built using Cytoscpape, shows gene ontology (GO) terms significantly enriched (false discovery rate < 0.25) in terms of cellular components, biological pathways and molecular functions in GSEA (males on the left and females on the right). Groups with fewer than 5 gene sets were deleted. *n* = 5 WC females; *n* = 5 WH females; *n* = 4 WC males; *n* = 4 WH males.

Cluster analysis allowed the identification of the biological functions of the enriched gene sets (Figure 6). In males, there are two large clusters with gene sets associated with postsynaptic component synaptic and dynein motile cilium. There are also seven other clusters with a lower number of enriched gene sets, including five linked to metabolic processes (complex iv mitochondrial, cholesterol efflux lipid, catabolic process acid, glutathione metabolic thyroid and metal ion homeostasis) and two linked to immunity (antigen mhc processing and suppression release host). Apart from the synapse-related cluster, all gene set clusters are increased by the maternal HF. In 7-month-old females, a large cluster of gene sets associated with mitochondrial ribosomal subunit is increased by the maternal HF. In addition to this cluster, four gene set clusters are enriched: two are decreased (component presynaptic active and voltage gated channel) and two are increased (cell regulation positive and endopeptidase peptidase regulator). Interestingly, although some sex-specific gene sets differences are observed, such as the cilium found only in males, commonalities are also present. The synapse appears to be affected in both males and females, with a reduction in related gene sets by the maternal HF. Mitochondria were also affected in both males and females, with related gene sets increased by the maternal HF. Notably, 20 mitochondria-related gene sets are enriched in both males and females, with a higher significance (FDR q-value) in females (Figure 5B and Figure 6). In addition, leading edge analysis (LEA) of common mitochondria-related gene sets between males and females, which allowed the identification of the genes mostly involved in enriching these gene sets, indicated that several genes involved in oxidative phosphorylation complexes, are key targets of the maternal HF, in particular in females. Taken together, these data indicate that the maternal HF significantly modifies the hippocampal transcriptome of 7-month-old male and female offspring with particular alterations of mitochondrial and synaptic pathways.

### 3.6. Identification of Upstream Regulators Triggering Changes in Gene Expression Induced by Maternal High-Fat Diet

**Figure 7:**
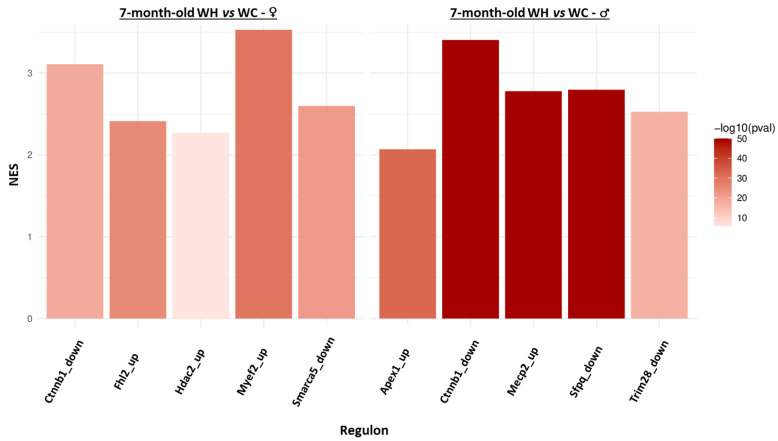
Identification of regulons deregulated by the maternal high-fat diet from hippocampal transcriptome and proteome data of 7-month-old male and female offspring. The bar plots represent regulons significantly (adjusted *p*-value < 0.001 and |normalized enrichment score (NES)| > 2) enriched in upregulated/downregulated genes in WH mice (females on the left and males on the right). A positive NES means that the regulon is enriched in upregulated genes. In the regulon name, “up” and “down” correspond to the activating or inhibiting effect of the transcription factor on the regulon genes, respectively.

Since it is now widely accepted that transcriptome and proteome changes are not always correlated in response to environmental modifications, we also performed analysis of the hippocampal proteome of 7-month-old male and female offspring by tandem mass spectrometry (using the same animals for RNAseq and mass spectrometry studies). GSEA analysis indicated that the maternal HF modifies very few enriched protein sets in 7-month-old offspring, with two clusters in males (motive atp respiration increased and negative regulation actin decreased) and none in females (Supplementary Data S3). In order to get an overview and to integrate hippocampal transcriptomic and proteomic data, we studied the regulome of 7-month-old offspring. Briefly, the aim of this approach is to identify regulons (a group of genes that co-vary and are regulated by the same transcription factor) and finally to identify whether each identified regulon is associated with the maternal HF. Five regulons are found respectively deregulated by the maternal HF in males (Apex1, Ctnnb1, Mecp2, Sfpq, Trim28) and females (Ctnnb1, Fhl2, Hdac2, Myef2, Smarca5), and only one is common to both sexes (Ctnnb1) (Figure 7). Interestingly, all these regulons exhibit increased expression of their genes by the maternal HF (NES > 0).

**Figure 8:**
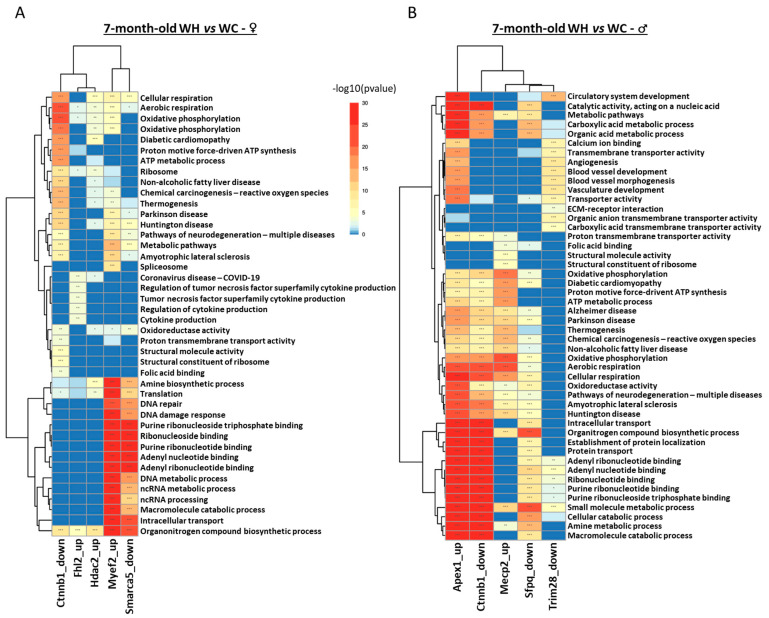
Analysis of pathway enrichment in regulons associated with maternal high-fat diet in 7-month-old male and female offspring. (**A**,**B**) The heatmap represents the top 5 gene ontology terms (biological processes, molecular functions and Kyoto encyclopedia of genes and genomes (KEGG)) enriched in each of the regulons in Figure 7, respectively, in male and female offspring. * *p* < 0.05, ** *p* < 0.01, *** *p* < 0.001.

Pathway enrichment analysis indicated that, although males and females have only one common regulon deregulated by maternal HF, the other regulons are associated with similar functions in both sexes (Figure 8). In males, the Mecp2, Apex1 and Sfpq regulons are associated with metabolic processes, cellular respiration, and RNA-related processes. In females, the Myef2, Fhl2 and Smarca5 regulons are also associated with these functions. The Ctnnb1 regulon, which is deregulated in both sexes, is involved in metabolic processes and cellular respiration. However, there are also differences: blood vessels appear to be affected only in male offspring (Mecp2 and Trim28 regulons), while cytokine production is deregulated only in females (Hdac2 regulon), suggesting sexual dimorphism.

## 4. Discussion

It is now widely accepted that, according to the concept of the developmental origin of health and diseases (DOHaD) [7], a maternal high-fat diet (HF) during the early stages of life induces long-lasting changes, such as metabolic [49] and cognitive dysfunctions [15]. Indeed, the perinatal period is a critical window for brain development with peak maturation occurring during lactation in rodents, which corresponds to the last trimester of gestation in humans. As a result, this period is particularly sensitive to environmental stresses [29]. At present, numerous studies investigating the consequences of a maternal HF on the central nervous system have demonstrated the deleterious effects on the hypothalamus to explain metabolic disorders [49,50,51]. On the other hand, few studies have examined the impact on the hippocampus, even though this structure is sensitive to metabolic disorders and is involved in cognitive processes [18]. Indeed, the hippocampus represents a central computing center in the brain, capable of detecting and responding to various stresses [52]. Some recent studies have investigated the impact of a maternal HF on hippocampal synaptic plasticity [19,22,24,45,53], neuroinflammation [54], and on cognition [16,17]. However, they did not combine integrated experimental approaches, such as behavioral tasks and a multi-omics strategy, nor did they take into account the sexual dimorphism that has been demonstrated to exist in the brain [55,56], as well as in response to perinatal environmental changes [21,26,27,28]. The present study focused on the effects of maternal HF during lactation in the young adult male and female offspring and examined, via a multi-omics approach, the integrated consequences on hippocampal transcriptome, proteome and regulome in order to identify early programming mechanisms putatively involved in cognitive disorders.

At the metabolic level, we show that a maternal HF during lactation decreases the body weight of dams and increases that of the offspring at weaning, suggesting an “energy transfer”. These results are in line with a recent meta-analysis of animals indicating that a maternal HF increases body weight of offspring at weaning [57]. Several studies suggest that this effect is due to richer milk and/or increased milk consumption caused by modified maternal behavior [58,59]. In our study, the increased body weight observed in weaned mice from maternal HF was normalized in adulthood, whereas it is usually still augmented in other reports probably reflecting strong heterogeneity between studies [57]. The absence of long-term effects of maternal HF on offspring body weight is correlated with normal concentrations of plasma triglyceride, cholesterol and free-fatty acids. In addition, maternal HF did not modify fasting glycaemia as well as body temperature, suggesting that in 7-month-old animals, maternal HF has few consequences on offspring metabolic parameters. However, male offspring from dams that were fed a HF failed to respond to a supra-physiological glucose load suggesting mild glucose intolerance presumably due to altered insulin secretion. This effect is consistent with the decrease in insulin-fasting levels in this experimental group. However, since we performed the glucose tolerance test at 3 months of age and measured insulin levels at 7 months of age, future studies are clearly required to better analyze sex-dimorphic effects of maternal HF on glucose homeostasis in the offspring. Indeed several studies have also reported disturbances in glucose homeostasis induced by a maternal HF [22], whereas others show no change [60] or a beneficial effect of the diet [27]. Moreover, the mild glucose intolerance observed only in males runs counter to Daerden and Balthasar’s study showing that a maternal high-fat/high-sugar diet during the perinatal period induces glucose intolerance only in female offspring [61]. These apparently contradictory results may be explained by different diet application periods, strains and diet compositions, but also by the age of the offspring. It is possible that glucose homeostasis alterations would appear in older animals in particular if they are still fed by HF after weaning [62].

In humans, a meta-analysis indicates that maternal obesity induces neurodevelopmental impairments leading to cognitive disorders (attention deficit hyperactivity disorder, autism spectrum disorder, and emotional and behavioral problems) in the offspring [15]. Similarly, a recent study of a cohort of 11,276 children associated an increase in body mass index in pregnant women with a decrease in various cognitive scores in the adolescents [63]. In line, several studies applying a maternal HF before mating, during gestation, and lactation show impaired long-term spatial memory in adult offspring [23,45,46]. This effect is long-lasting and can be passed on to subsequent generations [22]. Accordingly, we show here that the maternal HF during lactation impairs long-term spatial memory in male and female offspring, without locomotor or anxiety disorders, indicating that application of maternal HF during a shorter period (lactation) is sufficient to induce memory alterations. This also suggests that the hippocampus is a sensitive and early target of maternal HF in the offspring.

To date, although it has been published that maternal HF exerts long-lasting cognitive impairments associated with synaptic changes in the hippocampus of adult offspring [19,22,45,53], no global, unbiased and integrative approaches have been reported. Using a multi-omics approach, combining hippocampal transcriptome and proteome analysis of 7-month-old male and female mice, we show that there is a profound change in the hippocampal transcriptome in 7-month-old animals, when cognitive decline is observed. The gene sets decreased by the maternal HF in males and females are mainly associated with synaptic processes, and those increased with mitochondrial processes. However, the genes involved in the enrichment of these gene sets are different according to sex, suggesting that although the biological consequences are similar, the genes responsible for this deregulation are different between males and females. Such sex-specific divergent trajectories of gene expression have already been observed in the placenta of mice from maternal HF [64]. The biological mechanisms involved in sex-specific effects of maternal HF are not fully understood but may result from sex hormones that can modify the developmental trajectory and the susceptibility to environmental insults [28]. Future studies will need to investigate the way by which maternal HF programs gene expression in a sex-dependent manner. The decrease in synaptic processes is in line with studies showing a cognitive decline. Indeed, it has been reported that an obesogenic diet induces a loss of dendritic spines and alterations in their morphology in the adult offspring hippocampus [22,45,53,65], and decreases synaptic protein expression [19]. As synapses play a fundamental role in memory mechanisms, their loss could be at the root of the observed cognitive declines. Interestingly, transcriptome analysis of total fetal brain showed that a maternal HF also induced deregulation of genes associated with synapse organization, suggesting that synaptic changes in adulthood may be the result of modifications during neurodevelopment [66]. More surprising is the increase in mitochondrial-related gene sets by the maternal diet. Indeed, it is widely accepted that mitochondrial processes decline during aging [67,68], neurodegenerative diseases [69], or cognitive impairment [70]. Similarly, several studies indicate that a maternal HF can program a later decrease in brain mitochondria function [71,72], although no studies have been carried out in the hippocampus. Conversely, an increase in mitochondrial function, by a physical exercise for example, is associated with positive consequences, such as memory improvement [73]. However, a study on the regulation of the oxidative phosphorylation chain during aging indicates that complexes I, II, IV, and V are decreased at 24 months of age in mice, but that they are increased at 12 and 18 months of age [74]. The authors suggest that an adaptive mechanism is put in place to compensate for brain dysfunctions such as synaptic loss, which is also reported in a second study [75]. In fact, in a healthy young individual, the increase in the oxidative phosphorylation chain increases ATP production, which in turn increases neurotransmitter release and memory processes [76]. It is thus plausible that the increased mitochondrial activity in young adults from maternal HF occurs to limit the brain dysfunction and synapses alterations. One could speculate that mitochondrial activity would decrease in the long-term, promoting accelerated hippocampal aging and cognitive dysfunctions. In addition to the biological pathways impacted by the maternal HF in both males and females, others are sex-dependent. Among these pathways, gene sets associated with the cilium are increased by the diet only in male offspring. The primary cilium corresponds to an extension of the plasma membrane and has a primarily sensory role. In the brain, it is found in neurons and astrocytes, where it plays a role in regulating metabolism [77] and cognition [78]. Moreover, the primary cilium is also required during neurogenesis. Indeed, ablation of the primary cilium reduces the formation and proliferation of neural stem cells in the dentate gyrus, and alters the maturation of newly formed neurons [79]. Thus, an increase in primary cilium-related pathways could enhance neurogenesis, which may represent an adaptive mechanism to limit hippocampal alterations. In line with this, in the present study, we have also shown that the maternal HF during lactation increases adult hippocampal neurogenesis (AHN) only in male offspring. However, as with the increase in mitochondrial function, this result may seem surprising. Indeed, it has been reported in a mouse model of Alzheimer’s disease that neural stem cell ablation improves memory, suggesting a beneficial effect of AHN on cognitive processes [80]. In this sense, it has also been shown that the increase in AHN by physical exercise [81] or in transgenic mouse models [82] reduces age-related cognitive decline. In addition, studies indicate that a maternal HF during the perinatal period decreases cell proliferation and differentiation during AHN, altering memory [83,84]. However, other studies agree with our results, showing an increase in the number of stem cells and newly formed neurons in offspring whose mothers were fed a HF. In contrast, the authors show a decrease in the number of mature neurons [47], which is also reported in chronic early stresses [85]. This study suggests the importance of studying the maturation phase of newly formed neurons, which could be the subject of future work. Indeed, studies indicate that neurons with maturation defects do not function properly and cannot participate in the cognitive process. On the other hand, the increase in AHN could, in the same way as for mitochondria, be a compensatory mechanism in response to stress, but not sufficient to maintain functional cognitive processes. Moreover, this notable effect on AHN is present only in male offspring, suggesting sexual dimorphism which, to our knowledge, has never been shown in this context. These data need to be clarified, as gender-specific effects on neurogenesis vary widely depending on the study, the stress applied and the age of the animals.

The GSEA analysis suggests that the maternal HF has a small effect on the proteome, reinforcing the idea that there is therefore no correlation between the transcriptome and the proteome [86]. Nevertheless, proteomics data combined with transcriptomics data enabled us to develop a more integrative approach by performing a regulome analysis. This enabled us to identify regulators (transcription factors) deregulated at protein level by the maternal HF, which are associated with a modified expression of a group of genes known as regulon. In 7-month-old animals, the maternal HF induced a significant increase in several regulons in sex-specific manner. Interestingly, although the regulons differ according to sex, the associated biological functions are similar. Indeed, the regulons are mainly involved in mitochondrial respiration and the purine pathway. Interestingly, mitochondria supply precursors for purine de novo biosynthesis suggesting that the increase in mitochondrial function may participate in enhancing the purine pathway [87]. Future studies will be clearly required to analyze the consequences of maternal HF on the hippocampal mitochondria functionality and respiration in the offspring [88]. Moreover, these analyses allow us to target upstream regulators that could be interesting targets to better understand how the maternal HF modifies mitochondrial processes. Furthermore, it should be noted that only one regulon is common between the two sexes: Ctnnb1. Nevertheless, analyses of the top 50 most deregulated genes in this regulon indicates that none are in common between the two sexes, suggesting that gene deregulations differing according to sex can have the same consequences. Finally, this analysis also reveals differences between the sexes, with regulons deregulated by the diet associated with vascularization in males (Mecp2 and Trim28) and with cytokine production in females (Hdac2).

## 5. Conclusions

In summary, our results indicate that the application of a maternal HF only during lactation induces long-term memory impairment in adult mice offspring, associated with mild glucose intolerance only in males. Furthermore, the multi-omics and integrative approach (transcriptomics, proteomics, and regulomics) enabled us to show that hippocampal synaptic and mitochondrial processes are impacted by the maternal HF in both male and female offspring. However, although the biological consequences seem similar, the genes and proteins deregulated upstream appear to differ between the sexes. Finally, this approach, which has never before been carried out in this context, enables us to demonstrate that the hippocampus of the offspring is a sensitive and early-affected target of maternal HF. In addition, the effects of maternal HF reported here may help to better characterize sex-dependent molecular pathways involved in cognitive disorders and neurodegenerative diseases.

## Figures and Tables

**Table 1:** Effect of maternal high-fat diet during lactation on physiological and metabolic parameters in male and female offspring.

Parameters	WC 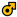	WH 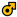	Significance	WC 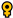	WH 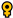	Significance
**Body weight at P21 (g)**	**8.6 ± 0.3**	**9.7 ± 0.3**	*p* = 0.010; **	**8.2 ± 0.2**	**10.0 ± 0.2**	*p* = 0.001; ***
**Body weight at the sacrifice—7 months (g)**	**34.3 ± 0.7**	**30.4 ± 1.0**	*p* = 0.006; **	24.2 ± 0.7	23.4 ± 0.8	*p* = 0.765
Glycemia fasting (6 h) —7 months (mg/dL)	166.7 ± 5.5	157.2 ± 6.8	*p* = 0.284	154.8 ± 4.8	151.4 ± 6.2	*p* = 0.666
**Insulinemia fasting (6 h)** **—7 months (µg/L)**	**0.7 ± 0.05**	**0.5 ± 0.07**	*p* = 0.030; *	0.3 ± 0.04	0.3 ± 0.01	*p* = 0.128
Free fatty acid—7 months (mmol/L)	0.2 ± 0.03	0.1 ± 0.04	*p* = 0.862	0.2 ± 0.02	0.2 ± 0.02	*p* = 0.594
Triglyceride—7 months (mg/dL)	53.7 ± 10.5	64.0 ± 7.3	*p* = 0.431	45.9 ± 3.4	48.9 ± 2.9	*p* = 0.578
Cholesterol—7 months (mg/dL)	61.7 ± 10.1	65.3 ± 7.9	*p* = 0.788	50.1 ± 3.7	47.7 ± 3.5	*p* = 0.659
Temperature (°C)	37.6 ± 0.1	37.5 ± 0.1	*p* = 0.540	37.4 ± 0.1	37.3 ± 0.1	*p* = 0.693

Values are represented as mean ± SEM. * *p* < 0.05, ** *p* < 0.01, *** *p* < 0.001 vs. WC mice and respective sex using Student’s unpaired *t*-test. Significant differences are indicated in bold. *n* = 19 WC females; *n* = 19 WH females; *n* = 17 WC males; *n* = 17 WH males.

## Data Availability

The RNA-sequencing data presented in this study are available in NCBI’s Gene Exoression Omnibus (GEO) database (accession number GSE167123). Mass spectrometry data presented in this study are available from the ProteomeXchange Consoritum via the PRIDE partner (accession number PXD045639).

## Supplementary Materials

The following supporting information can be downloaded at: https://www.mdpi.com/article/10.3390/nu15214691/s1, Table S1: Diet composition. Percentages of protein, fat and carbohydrates in each diet, and associated total amount of kilocalories per kilogram of body weight. Supplementary Data S1: Body weight and food intake of dams during lactation. (A) Body weight of dams that fed a chow (C) or high-fat (H) diet during lactation at different post-natal day (P1, P8, P15 and P21). (B) Food intake of dams that fed a C or H diet during lactation at different post-natal day (P8, P15 and P21). Values are represented as mean ± SEM. ** *p* < 0.01, *** *p* < 0.001 vs. C mice using two-way ANOVA followed by Tukey’s post hoc test. Supplementary Data S2: Effect of maternal high-fat diet during lactation on locomotion (A–D) and anxiety-like behavior (E,F) in 6-month-old male and female offspring, using respectively, actimetry and elevated plus maze. (A,B) Distance moved during the actimetry, respectively in male and female offspring. (C,D) Velocity during the actimetry, respectively in male and female offspring. (E,F) Percentage of time spent in the open arms, respectively in male and female offspring representing. *n* = 13 WC females; n = 11 WH females; *n* = 6 WC males; *n* = 10 WH males. Supplementary Data S3: Network analysis of gene set enrichment analysis (GSEA) results from the hippocampal proteomic of 7-month-old male and female offspring. The network, built using Cytoscpape, shows gene ontology (GO) terms significantly enriched (false discovery rate < 0.25) in terms of cellular components, biological pathways and molecular functions in GSEA (males on the left and females on the right). Groups with fewer than 5 gene sets were deleted.
