# Supplementary material for: Multi-Omics Data Integration Reveals Sex-Dependent Hippocampal Programming by Maternal High-Fat Diet during Lactation in Adult Mouse Offspring
Source: Nutrients. 2023 Nov 5;15(21):4691. doi: 10.3390/nu15214691 (PMC10649590; doi:10.3390/nu15214691)
Supplement: Supplementary file 1 [file nutrients-15-04691-s001.zip › nutrients-2673931-supplementary.pdf]

Supplementary Material:

Actimetry

Mice were placed in the center of an infrared actimeter (45x45x35 cm; Bioseb), composed by a 2-dimensional square frame, and left for 10 min. Spontaneous behavior of mice was tracked with distance moved and velocity recorded by Actitrack software (Bioseb).

Elevated Plus Maze

The elevated plus maze (EPM) was used to investigate anxiety-like behavior. The apparatus consists of a plus-shaped maze with two closed and two open arms (30 cm long x 6.5 cm wide). Mice were placed at the center of the maze with their face in the direction of a closed arm and were allowed to explore freely for 5 min. Time spent in open arms was recorded using Ethovision XT tracking system (Noldus).

Table S1. Diet composition. Percentages of protein, fat and carbohydrates in each diet, and associated total amount of kilocalories per kilogram of body weight

	**Control diet**	**High-fat diet**	**Standard diet**
Period	P0 until P1		P21 until sacrifice
Reference	SAFE A03	Research diets D12331	SAFE A04
% protein	25.2	16.5	19.3
% lipid	13.5	58.0	8.4
% carbohydrates	61.3	25.5	72.4
Kcal/Kg	3395	5558.5	3339

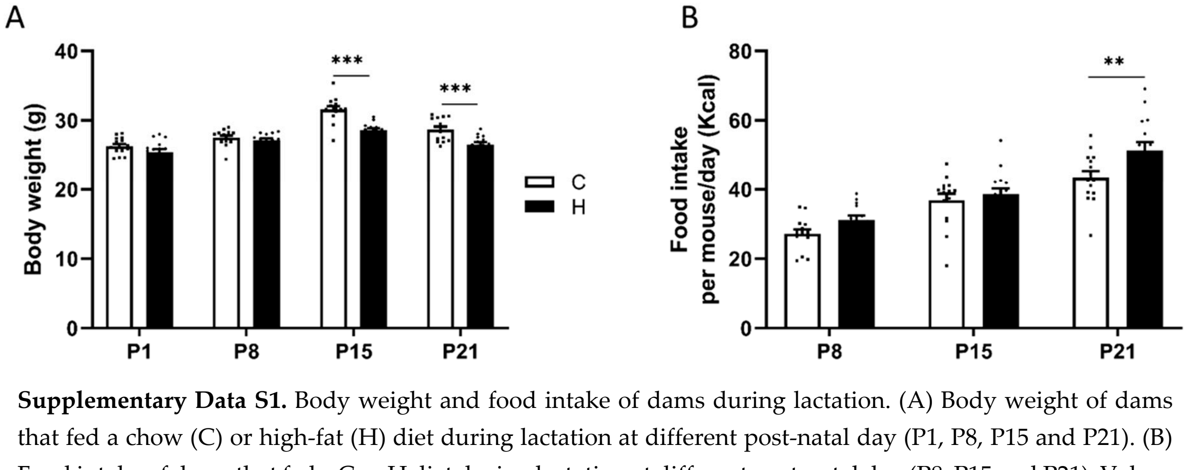

Supplementary Data S1. Body weight and food intake of dams during lactation. (A) Body weight of dams that fed a chow (C) or high-fat (H) diet during lactation at different post-natal day (P1, P8, P15 and P21). (B) Food intake of dams that fed a C or H diet during lactation at different post-natal day (P8, P15 and P21). Values are represented as mean ± SEM. **p < 0.01, ***p < 0.001 *vs* C mice using two-way ANOVA followed by Tukey's *post hoc* test.

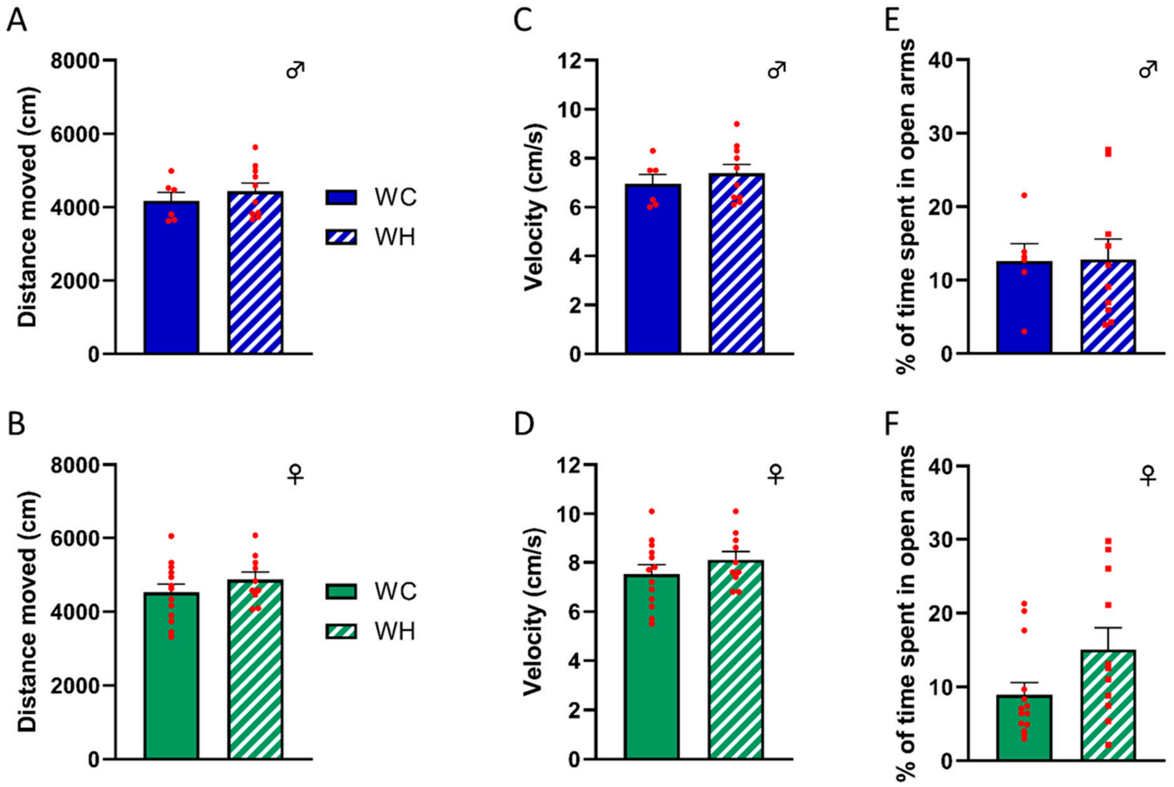

Supplementary Data S2. Effect of maternal high-fat diet during lactation on locomotion (A-D) and anxiety-like behavior (E-F) in 6-month-old male and female offspring, using respectively, actimetry and elevated plus maze. (A-B) Distance moved during the actimetry, respectively in male and female offspring. (C-D) Velocity during the actimetry, respectively in male and female offspring. (E-F) Percentage of time spent in the open arms, respectively in male and female offspring representing. n=13 WC females; n=11 WH females; n=6 WC males; n=10 WH males.

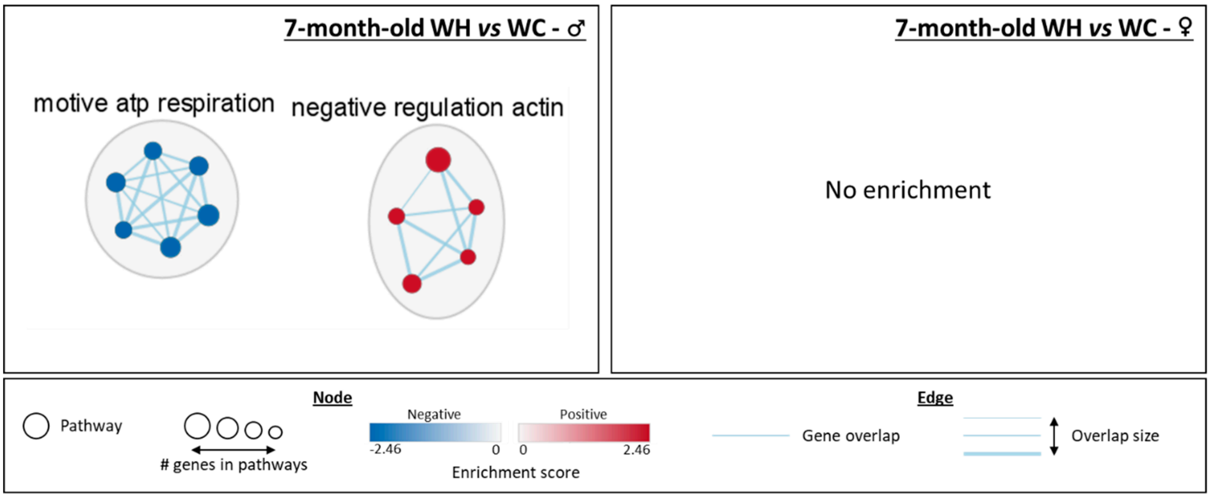

Supplementary Data S3. Network analysis of gene set enrichment analysis (GSEA) results from the hippocampal proteomic of 7-month-old male and female offspring. The network, built using Cytoscpape, shows gene ontology (GO) terms significantly enriched (false discovery rate < 0.25) in terms of cellular components, biological pathways and molecular functions in GSEA (males on the left and females on the right). Groups with fewer than 5 gene sets were deleted.
